# Some Points of Preventive Treatment in the Diseases of Women

**Published:** 1899-06-10

**Authors:** Arthur E. Giles

**Affiliations:** Assistant Surgeon Chelsea Hospital for Women


### HOSPITAL CLINICS AND MEDICAL PROGRESS.

### SOME POINTS OF PREVENTIVE TREATMENT IN THE DISEASES OF WOMEN.

By ARTHUR E. GILES, M.D., B.Sc., M.R.C.P.Lond., F.R.C.S.Ed., Assistant Surgeon Chelsea Hospital for Women.

**Diseases Resulting from Child-bearing.**—(*Continued from p.* 162.)—The third suggestion is that when operative interference is necessary this should be carried out with the utmost care. While cases still occur in which the uterus or vagina is much damaged, in which one blade of the forceps is applied outside the cervix, in which the perforator is thrust through the uterus, or the bladder is injured, this suggestion cannot be regarded as gratuitous. Further, just as it is bad practice to interfere unnecessarily, so, also, it is bad to wait too long before interfering. When the head is left for a considerable time wedged in the pelvis, whether at the brim, in the cavity, or at the outlet, serious damage may result to the structures pressed upon, so that the vaginal wall or the cervix may slough, and pelvic inflammation supervene, or a vesico-vaginal fistula form; hæmatoma of the vulva may occur; and the perineum may become so friable that a considerable rupture is inevitable. Besides these complications, which may be looked on as preventable after-results, there are also immediate dangers due to delay, such as exhaustion and collapse.

One more suggestion is necessary, and that is that proper care be exercised during the lying-in period. The effect of a too early resumption of duties, and even of getting up too early, is a predisposition to sub-involution; this leads in turn to a series of more or less troublesome disturbances—retroversion of the uterus, chronic engorgement, chronic endometritis, with their symptoms of pain, leucorrhœal discharges, and general weakness.

In spite of the most careful attention confinement is sometimes followed by one or more of the troubles above indicated; but a large proportion of them may be prevented, and it is for this reason that we invite the thoughtful consideration of all who are concerned with the care and treatment of "women labouring with child."

**The Prevention of Grave Disease by the Early Treatment of Slight Ailments.**—Patients not infrequently apologise to their medical attendants for consulting them for slight complaints. There is, however, no occasion for apology; it is rather an indication of wisdom, if there be any truth in the proverb, "Prevention is better than cure." In the department of diseases of women the tendency is towards the opposite extreme; and patients will often endure pain, discomfort and general ill-health for months, or even years, before seeking advice. There are obvious reasons why this should be so; but it is none the less a frequent disadvantage to both patient and doctor, for in many cases the treatment is rendered much longer and more difficult; and sometimes the disease reaches such an advanced stage that cure is no longer possible.

In laying down rules for the prevention of the diseases of women we must therefore include these two: (1) To patients—seek advice early; (2) to doctors—treat carefully slight ailments. I should like particularly to apply these rules to two symptoms, namely, hæmorrhage and offensive discharges.

**Hæmorrhage.**—When the menstrual periods become increased in quantity, as compared with the usual amount of blood lost; when this goes on for several consecutive monthly periods; and especially when losses of blood occur in the intervals between the periods, the patient should lose no time before seeking advice, and her medical attendant should at once carefully seek for the cause of the hæmorrhage.

I need only mention the principal conditions to which such loss of blood may be due. They are uterine polypus, certain forms of endometritis, fibro-myomatous tumours, miscarriage, threatened or incomplete, and cancer of the uterus. The continued and excessive loss of blood is in itself harmful, and may induce a condition of anæmia which it may take years to recover from. And when the cause is such as admits of early and thorough cure, it is a great pity that so much unnecessary ill-health should be allowed to develop. The causes which come under this heading are polypus, endometritis, and the retained portions of placenta in cases of miscarriage.

But still more serious are cases in which a fibromyoma or cancer is allowed to attain dangerous proportions before treatment is begun. The risk of such a calamity occurring is greatest at about the time of the "change of life." For irregular hæmorrhages often occur at this time, even when the organs are healthy; and, consequently, patients and doctors alike are sometimes thrown off their guard in cases where a cancer may be making fatal strides. There are few things more tragic for the patient, or more distressing to her medical adviser than the discovery of a cancerous growth too advanced to allow of cure; yet it is an experience all too common, both in private and in hospital practice. We cannot, therefore, too strongly urge the importance of seeking advice early, and the necessity for early and thorough examination when any abnormal symptoms are present. If no disease be found, no harm has been done by ascertaining the fact; if there be early disease, its discovery in time will never be regretted.

**Offensive Discharges.**—These may be due to endometritis, to a sloughing fibroid or polypus, to decomposing fragments of placenta left after a miscarriage, to a neglected or perhaps forgotten pessary, or to cancer. What we have said as to the importance of early recognition of the cause of hæmorrhage applies equally to the early diagnosis of the source of these discharges; for by such early diagnosis and treatment much suffering may be averted, and the patient may be saved from grave risk and restored to health and to life.

Passing on to other points, we may observe that much ill-health and discomfort often results from a torn cervix or perineum; the former, as previously stated, predisposes to endometritis, erosion, and their complications; whilst the latter leads to prolapse of the vaginal walls and uterus, and even to complete hernia of these organs outside the body. The prevention of these conditions is easily effected by repairing the torn structures. The necessary operation is in either case free from risk, and entails only two or three weeks of "lying-up"; at such a small cost it is worth while to avert the chronic disorders which result as the alternative.

Sometimes treatment by pessaries is adopted, to remedy the inconveniences of prolapse. When for any reason no radical treatment is admissible, pessaries may be employed as the next best thing; but we must still regard them as a necessary evil. We have all known patients go about, having worn pessaries for five, ten, or twenty years; and in many instances the unhealthy condition of their organs speaks eloquently against such a mode of treatment when a simple and effective procedure is possible, which will bring about a cure once for all. If the disadvantages of pessaries were duly recognised we should not meet with cases, as we do now, where a long-retained pessary has to be literally cut out from the structures which have grown over and around it.

